# Supplementary material for: Finding the Gaps: Integrated Serosurveillance and Spatial Clustering of Vaccine Preventable Diseases in Samoa, 2018–2019
Source: Trop Med Infect Dis. 2025 Dec 28;11(1):9. doi: 10.3390/tropicalmed11010009 (PMC12846106; doi:10.3390/tropicalmed11010009)

Supplementary Material S1:
Table S1: Vaccination schedule in Samoa (Adapted from Samoan Government., National Immunisation Policy 2020-2025, Ministry of Health, Editor. 2020: Samoa)

Supplementary Material S2: Cut-offs for seropositivity (Table S2) and figures showing cut-offs against the distribution of individual responses (Figure S1)

Table S2: Median fluorescence intensity - background (MFI-bg) Cut-offs for seropositivity by level of protection in International Units (IU) and batch number.

Figure S1: Histogram of individual MFI-bg[a] (log10) for A) measles (≥0.01 IU[b]) with cutoffs at 1361 (black line) for samples tested in batch 1[c] and 1533 (orange line) for samples tested in batch 2[d] ; B) rubella (≥0.01 IU[b]) with cutoffs at 1558 (orange line) for samples tested in batch 2[d] and 987 (black line) for samples tested in batch 1[c]; C) diphtheria with cutoffs at 386 for ≥0.01IU[b] (black line) and 7761 for ≥0.1IU[b] (orange line); D) tetanus with cutoffs at 100 for ≥0.01IU[b] (black line), 1523 for ≥0.1IU[b] (orange line), and 2372 for ≥1IU[b] (green line).
a-Median fluorescence intensity-background
b- International Units
c-Samples collected in 2018 and 2019
d-Samples collected in 2019 only

Supplementary Material S3: Gender and age-group seroprevalence estimates (Table S3) and histogram of age distribution (Figure S2).

Table S3: Gender and age-group seroprevalence estimates[a] (%) for ≤0.01 International Units to measles, rubella, diphtheria, and tetanus with 95% confidence intervals (CI) in Samoa 2018-2019.
a - Adjusted for study design

Figure S2: Histogram of age distribution

Supplementary Material S4:
Table S4: Primary sampling unit (PSU) level seroprevalence estimates[a] (%) with 95% confidence intervals (CI) for ≤0.01 IU[b] in Samoa 2018-2019.
a - Adjusted for study design and standardised to age and sex
b - International Units

Supplementary Material S5:
Table S5: Primary sampling unit (PSU) level seroprevalence estimates[a] (%) for ≤0.01 IU[b] in Samoa by year (2018, 2019).
a - Adjusted for study design and standardised to age and sex
b - International Units

Supplementary Material S6: Characteristics of participants seronegative to all VPDs (measles, rubella, diphtheria, and tetanus).

Table S6: Overall (n=102) demographics of participants seronegative to all VPDs (measles, rubella, diphtheria, and tetanus).

Figure S3: Map of primary sampling unit (PSU) level seroprevalence estimates[a] (%) for seronegativity to all vaccine preventable diseases (measles, rubella, diphtheria, and tetanus) in Samoa 2018 & 2019. This figure was created using ArcGIS Pro (Version 3.1.0).

[a] - Adjusted for study design and standardised to age and sex

Figure S4: Map of household GPS coordinates and sampled primary sampling units.

Figure S5: Overlap of Map of primary sampling unit (PSU) level seroprevalence estimates[a] (%) for seronegativity to all vaccine preventable diseases (measles, rubella, diphtheria, and tetanus) with household GPS coordinates and spatial cluster of seronegativity to measles.

[a] - Adjusted for study design and standardised to age and sex

Supplementary Material S1:

Table S1: Vaccination schedule in Samoa (Adapted from Samoan Government., National Immunisation Policy 2020-2025, Ministry of Health, Editor. 2020: Samoa)

Age	Vaccine/s	Disease/s covered
**6 weeks**	DTPa<br>HepB<br>HiB<br>bOPV<br>Rotarix	Diphtheria, Tetanus and Pertussis<br>Hepatitis B<br>Haemophilus influenzae B<br>Poliomyelitis<br>Rotavirus
**10 weeks**	DTPa<br>HepB<br>HiB<br>bOPV<br>Rotarix	Diphtheria, Tetanus and Pertussis<br>Hepatitis B<br>Haemophilus influenzae B<br>Poliomyelitis<br>Rotavirus
**14 weeks**	DTPa<br>HepB<br>HiB<br>bOPV	Diphtheria, Tetanus and Pertussis<br>Hepatitis B<br>Haemophilus influenzae B<br>Poliomyelitis
**9 months**	MMR<br>TCV	Measles, Mumps and Rubella<br>Typhoid
**15 months**	MMR<br>DTP<br>OPV	Measles, Mumps and Rubella<br>Diphtheria, Tetanus and Pertussis<br>Poliomyelitis
**5 years**	Td booster	Tetanus and Diphtheria
**10 years**	Td booster	Tetanus and Diphtheria
**Expectant mothers**	Td booster	Tetanus and Diphtheria

Supplementary Material S2: Cut-offs for seropositivity (Table S2) and figures showing cut-offs against the distribution of individual responses (Figure S1)

Table S2: Median fluorescence intensity - background (MFI-bg) Cut-offs for seropositivity by level of protection in International Units (IU) and batch number.

VPD	Year	Level	Cutoff (MFI-bg)	Batch	Source
**Measles**	2018	≥0.01 IU	1361	NA	JR SC 10/2021
	2019		1361	1	
			1533	2	4/14/23 EE
**Rubella**	2018	≥0.01 IU	987	NA	JR SC 10/2021
	2019		987	1	
			1558	2	3/15/23 BG
**Diphtheria**	2018	≥0.01 IU	386	NA	JR SC 10/2021 And 3/2/22 GC
		≤0.1 IU	7761		
	2019	≥0.01 IU	386		
		≤0.1 IU	7761		
**Tetanus**	2018	≥0.01 IU	100	NA	JR SC 10/2021
		≤0.1 IU	1523		
		≤1 IU	23272		
	2019	≥0.01 IU	100		
		≤0.1 IU	1523		
		≤1 IU	23272		

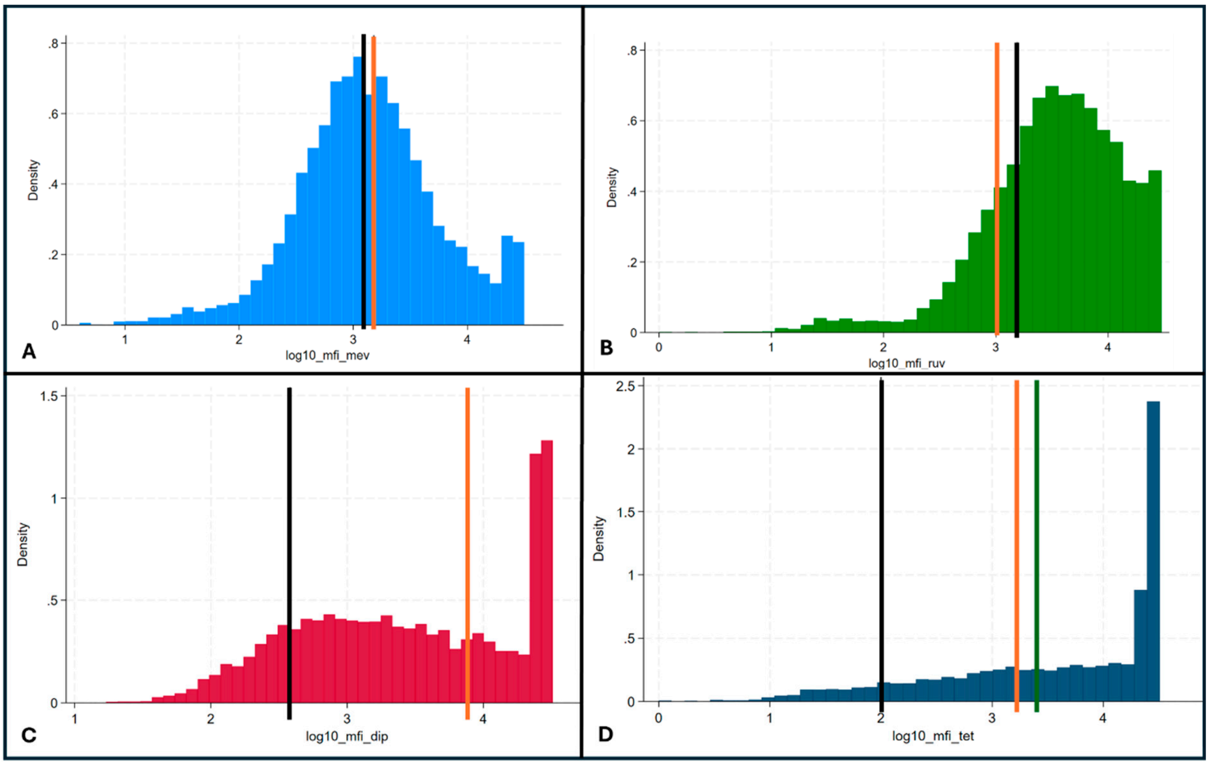

Figure S1: Histogram of individual MFI-bg[a] (log10) for A) measles (≥0.01 IU[b]) with cutoffs at 1361 (black line) for samples tested in batch 1[c] and 1533 (orange line) for samples tested in batch 2[d] ; B) rubella (≥0.01 IU[b]) with cutoffs at 1558 (orange line) for samples tested in batch 2[d] and 987 (black line) for samples tested in batch 1[c]; C) diphtheria with cutoffs at 386 for ≥0.01IU[b] (black line) and 7761 for ≥0.1IU[b] (orange line); D) tetanus with cutoffs at 100 for ≥0.01IU[b] (black line), 1523 for ≥0.1IU[b] (orange line), and 2372 for ≥1IU[b] (green line).

a-Median fluorescence intensity-background

b- International Units

c-Samples collected in 2018 and 2019

d-Samples collected in 2019 only

Supplementary Material S3: Gender and age-group seroprevalence estimates (Table S3) and histogram of age distribution (Figure S2)

Table S3: Gender and age-group seroprevalence estimates[a] (%) for ≤0.01 International Units to measles, rubella, diphtheria, and tetanus with 95% confidence intervals (CI) in Samoa 2018-2019.

a - Adjusted for study design

Gender	Age group	Seroprevalence (95% CI)			
		Measles	Rubella	Diphtheria	Tetanus
Male	All ages	44.0 (42.4-45.7)	78.6 (77.0-80.2)	82.6 (81.0-84.1)	90.8 (89.6-91.9)
	5-9 years	40.0 (38.0-42.1)	80.1 (78.4-81.7)	81.9 (80.3-83.5)	90.2 (88.9-91.4)
	10-19 years	23.9 (21.0-27.1)	67.3 (63.6-70.8)	85.6 (82.6-88.1)	86.6 (83.7-89.0)
	20-29 years	16.5 (12.8-21.0)	84.2 (79.7-87.8)	79.5 (74.7-83.6)	89.3 (85.4-92.3)
	30-39 years	42.4 (36.0-48.9)	82.8 (77.2-87.2)	84.0 (78.5-88.3)	94.9 (91.0-97.1)
	40-49 years	76.7 (70.7-81.8)	84.2 (78.8-88.5)	76.3 (70.3-81.4)	94.4 (90.5-96.7)
	50-59 years	82.3 (76.9-86.6)	79.0 (73.3-83.7)	81.9 (76.6-86.3)	95.3 (91.8-97.4)
	60-69 years	88.7 (83.0-92.7)	84.5 (78.3-89.1)	88.7 (83.0-92.7)	94.1 (89.3-96.8)
	70+ years	87.0 (78.8-92.3)	84.4 (75.8-90.4)	88.4 (80.6-93.3)	88.2 (80.1-93.3)
Female	All ages	47.8 (46.4-49.2)	80.1 (78.7-81.4)	84.9 (83.7-86.1)	91.2 (90.2-92.1)
	5-9 years	49.8 (47.6-52.0)	82.4 (80.6-84.0)	82.4 (80.6-84.0)	89.3 (87.9-90.6)
	10-19 years	26.4 (23.5-29.6)	71.3 (67.8-74.4)	83.0 (80.1-85.5)	85.0 (82.2-87.4)
	20-29 years	20.4 (17.2-24.1)	81.1 (77.5-84.2)	92.6 (90.0-94.6)	93.6 (91.1-95.4)
	30-39 years	40.6 (35.8-45.2)	82.1 (77.9-85.7)	90.1 (86.7-92.7)	96.2 (93.7-97.7)
	40-49 years	83.6 (79.1-87.1)	87.0 (82.8-90.3)	84.9 (80.6-88.4)	95.8 (92.9-97.5)
	50-59 years	89.8 (85.7-92.8)	86.0 (81.4-89.5)	73.3 (67.9-78.2)	95.9 (93.0-97.6)
	60-69 years	84.9 (78.9-89.4)	76.5 (69.7-82.1)	79.0 (72.5-84.3)	93.5 (88.8-96.3)
	70+ years	87.4 (80.0-92.4)	89.1 (81.8-93.7)	86.7 (79.3-91.7)	84.5 (76.5-90.1)

Figure S2: Histogram of age distribution

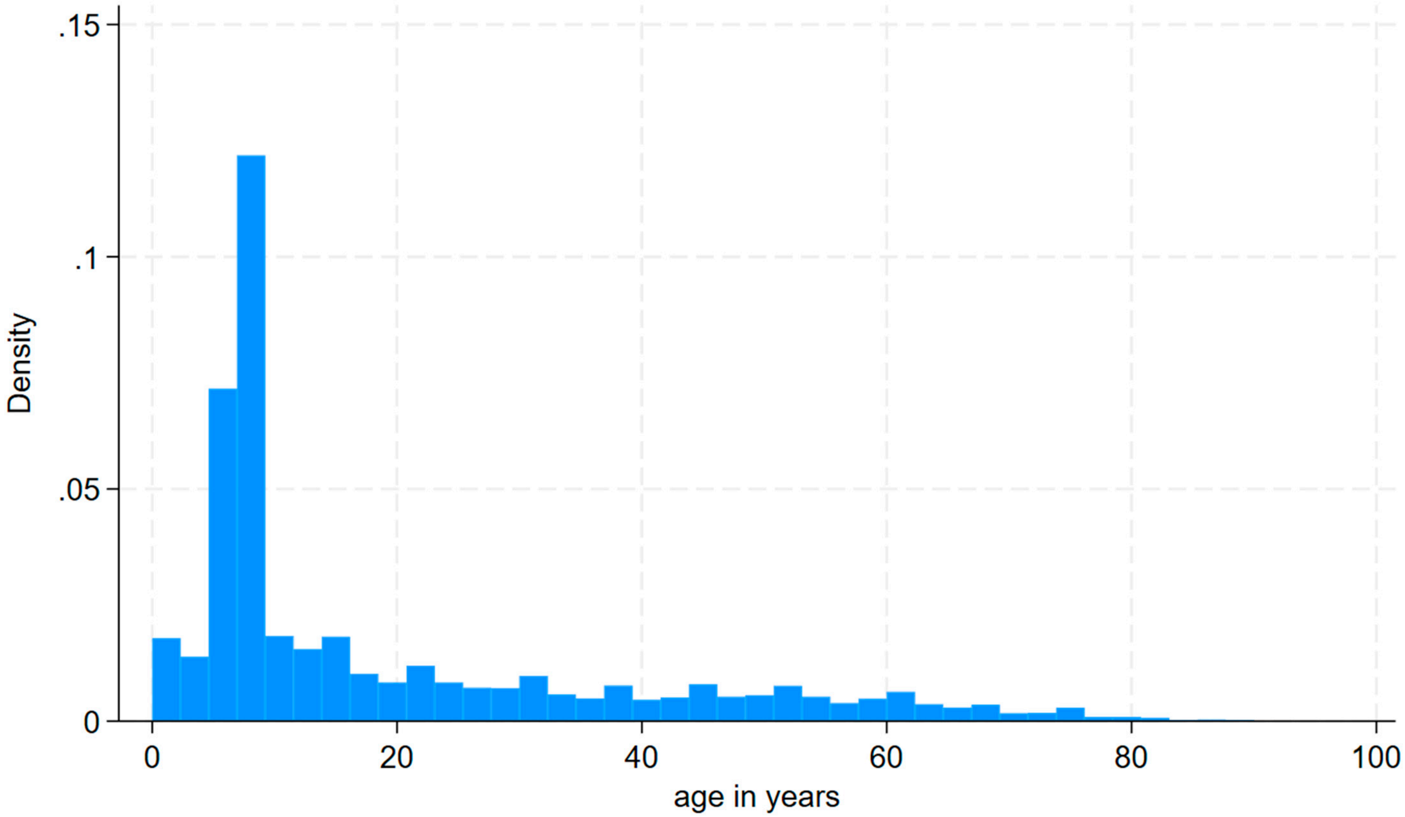

Supplementary Material S4:

Table S4: Primary sampling unit (PSU) level seroprevalence estimates[a] (%) with 95% confidence intervals (CI) for ≤0.01 IU[b] in Samoa 2018-2019.

a - Adjusted for study design and standardised to age and sex

b - International Units

Region	Primary Sampling Unit	Seroprevalence (95% CI)			
		Measles	Rubella	Diphtheria	Tetanus
**Apia Urban Area**	Vaivase Tai	50.9 (47.2-58.0)	88.8 (78.1-88.1)	79.3 (72.6-83.7)	92.3 (85.8-96.0)
	Vaiala Tai + Vaiala Uta	52.9 (48.5-58.5)	86.4 (80.3-92.4)	90.6 (86.9-93.2)	93.3 (88.9-95.9)
	Avele + Letava	50.9 (45.5-58.9)	76.9 (72.9-82.8)	85.3 (79.4-89.6)	92.4 (86.0-95.8)
	Fugalei + Vaimea	31.0 (27.6-38.5)	76.5 (68.3-81.3)	73.8 (67.2-79.3)	88.5 (84.0-91.7)
	Vaimoso	47.6 (43.0-54.4)	82.3 (76.1-86.4)	69.7 (63.7-74.7)	83.0 (77.1-87.6)
	Vaitoloa	44.7 (38.8-50.5)	81.8 (75.4-87.0)	78.6 (71.8-84.0)	89.9 (83.7-93.9)
**Northwest Upolu**	Letogo	39.9 (34.8-48.3)	76.3 (70.0-81.2)	85.1 (78.2-89.0)	90.3 (84.0-94.0)
	Vaiusu	49.6 (44.1-53.9)	83.0 (78.4-87.3)	77.8 (71.5-81.3)	89.3 (84.4-91.4)
	Puipaa	36.8 (32.3-41.2)	85.4 (79.9-90.5)	68.5 (61.2-73.5)	89.2 (83.2-92.8)
	Ulualoloa	35.3 (39.4-50.4)	72.0 (81.8-90.3)	89.0 (82.1-90.6)	87.3 (79.9-90.2)
	Vaitele Fou	38.4 (36.8-48.7)	71.2 (76.1-86.7)	84.4 (76.9-88.2)	94.5 (89.3-96.9)
	Lotosoa	52.1 (41.7-55.9)	82.5 (73.3-85.2)	79.2 (71.0-83.2)	93.0 (89.7-94.8)
	Nuu	38.3 (32.8-44.0)	78.9 (72.5-84.3)	85.6 (79.6-90.0)	92.6 (87.9-95.5)
	Tuanai	42.1 (37.7-49.9)	81.0 (76.2-87.4)	79.1 (73.2-82.7)	92.7 (86.9-95.1)

	Fasitoouta	45.7 (35.6-49.5)	83.5 (76.4-86.9)	87.7 (80.1-91.7)	94.5 (89.9-96.4)
	Vailuutai	37.8 (33.4-42.4)	85.6 (80.7-88.7)	85.8 (81.2-89.4)	92.3 (88.5-94.9)
	Leauvaa	37.0 (31.9-42.4)	74.8 (70.3-81.3)	85.6 (78.2-89.5)	90.7 (83.9-94.0)
	Fasitoo Tai	49.0 (43.6-56.2)	82.6 (77.4-87.1)	83.0 (76.2-87.4)	85.6 (80.1-89.3)
	Faleasiu	41.0 (37.1-46.3)	76.1 (71.4-80.8)	80.2 (74.6-83.8)	85.8 (81.6-89.2)
	Laulii	44.9 (41.4-50.8)	80.4 (75.6-85.1)	81.9 (77.1-85.8)	89.3 (84.7-92.5)
**Rest of Upolu**	Fusi	40.8 (37.8-45.5)	76.1 (71.7-82.0)	84.2 (80.0-87.3)	95.3 (91.7-97.3)
	Faleseela	46.0 (42.9-53.0)	71.4 (69.0-80.1)	84.8 (77.1-89.6)	88.3 (82.2-92.2)
	Manono Uta	54.1 (47.0-57.2)	88.2 (83.7-92.0)	89.9 (84.7-93.1)	89.6 (83.6-93.0)
	Salani + Utulaelae	36.7 (31.5-43.3)	73.5 (68.0-78.5)	83.7 (79.6-86.8)	91.1 (86.3-94.1)
	Mutiatele + Saleaaumua	49.4 (46.1-59.4)	80.3 (80.4-90.0)	88.3 (82.1-91.3)	97.5 (94.9-98.7)
	Falefa	38.0 (36.5-46.9)	66.5 (61.6-74.7)	77.2 (70.3-81.8)	90.0 (83.7-93.4)
	Faleapuna + Musumusu	41.5 (39.7-50.9)	77.1 (73.9-85.3)	88.2 (82.9-91.6)	93.9 (90.1-95.9)
	Salua (Manono Island)	43.7 (39.4-51.5)	86.8 (74.5-86.0)	86.0 (79.5-88.8)	90.3 (87.6-92.0)
**Savai'i**	Lalomalava + Safua	55.4 (49.8-64.1)	80.3 (76.6-86.7)	88.2 (83.7-91.0)	95.6 (92.2-97.3)
	Lano	50.2 (44.4-58.4)	84.6 (74.7-86.7)	94.1 (90.1-96.4)	95.0 (89.9-97.5)
	Safotu	49.3 (45.0-57.5)	77.1 (67.1-81.5)	88.6 (83.9-91.9)	94.0 (89.2-96.8)
	Sataua	49.2 (42.5-52.9)	73.4 (67.1-79.6)	90.0 (84.7-93.2)	91.9 (87.4-94.6)
	Sagone	47.5 (41.7-54.4)	85.2 (77.9-89.0)	88.4 (82.9-92.3)	95.5 (89.8-98.1)
	Papa + Tafua	49.6 (45.4-55.0)	76.0 (67.6-77.5)	83.4 (78.5-87.0)	86.1 (81.9-89.3)
	Salelolonga	51.8 (45.5-59.5)	76.6 (68.5-79.9)	84.5 (77.9-89.1)	88.5 (82.7-92.3)

Supplementary Material S5:

Table S5: Primary sampling unit (PSU) level seroprevalence estimates[a] (%) for ≤0.01 IU[b] in Samoa by year (2018, 2019).

a - Adjusted for study design and standardised to age and sex

b - International Units

Region	Primary Sampling Unit	Tetanus		Rubella		Measles		Diphtheria	
		2018	2019	2018	2019	2018	2019	2018	2019
Apia Urban Area	Vaivase Tai	92.64	95.76	85.88	58.09	42.54	30.49	84.57	87.26
	Vaiala Tai + Vaiala Uta	90.06	98.51	71.78	95.43	45.33	65.96	91.23	87.6
	Avele + Letava	95.47	91.46	74.37	84.33	38.17	54.84	88.01	81.99
	Fugalei + Vaimea	86.64	97.64	69.15	47.33	22.23	33.12	88.09	52.95
	Vaimoso	80.88	75.19	76.97	68.3	44.44	36.35	69.41	54.97
	Vaitoloa	87.63	92.78	84.92	75.91	47.32	34.76	81.94	78.89
Northwest Upolu	Letogo	88.59	99.48	77.74	48.21	40.26	29.78	83.81	96.82
	Vaiusu	92.58	86.67	81.34	82.46	46.07	50.58	71.98	75.47
	Puipaa	81.86	95.07	84.21	87.11	42.16	31.75	73.77	67.3
	Ulualoloa	89.73	72.91	85.82	91.67	41.69	26.74	89.05	81.71
	Vaitele Fou	97.37	89.94	79.75	91.64	42.51	37.47	78.12	87.51
	Lotosoa	90.85	92.26	83.73	78.92	43.38	45.13	81.63	74.13
	Nuu	92.97	91.57	80.21	80.94	41.48	34.61	87.49	80.97
	Tuanai	85.72	95.28	84.54	84.92	36.03	45.47	77.27	81.1
	Fasitoouta	95.2	91.11	85.52	75.58	43.36	41.8	92.91	80.85
	Vailuutai	89.1	92.24	86.83	83.18	29.34	39.3	86.63	84.32
	Leauvaa	90.8	96.62	77.19	71.42	33.1	37.45	87.21	88.84
	Fasitoo Tai	82.4	86.63	85.46	77.03	45.7	51.7	81.89	77.5
	Faleasiu	88.18	85.08	62.14	82.79	35.4	42.33	79.91	77.68
	Laulii	91.68	88.27	81.1	78.52	42.5	47.29	83.65	81.43
Rest of Upolu	Fusi	95.33	94.26	73.24	78.42	40.51	42.52	84.54	81.34
	Faleseela	90.62	86.8	78.52	76.28	53.38	47.2	89.85	80.89
	Manono Uta	91.14	92.95	86.99	87.74	39.21	55.26	93.54	89.11

	Salani + Utulaelae	89.76	80.92	65.04	73.74	43.89	18.53	86.98	74.4
	Mutiatele + Saleaaumua	99.1	97.36	81.13	83.72	40.92	43.78	82.94	81.84
	Falefa	86.97	92.77	64.96	73.74	41.6	40.05	77.52	81.68
	Faleapuna + Musumusu	100	100	87.98	75.5	30.03	30.25	100	100
	Salua (Manono Island)	95.08	100	81.08	100	37.11	78.16	95.74	67.98
**Savai'i**	Lalomalava + Safua	91.55	95.22	80.26	78.73	40.38	45.13	88.93	90.68
	Lano	95.48	100	65.4	82.19	46.56	31.38	89.36	100
	Safotu	89.86	86.3	80.6	77.65	40.79	45.6	89.27	85.87
	Sataua	93	90.36	75.55	74.2	42.24	55.13	92.74	88.94
	Sagone	93.11	98.88	84.86	86.44	46.95	44.8	93.06	78.19
	Papa + Tafua	93.63	91.33	65.6	63.03	35.5	36.75	87.89	81.71
	Salelolonga	89.57	92.09	54.81	80.59	34.3	49.9	78.3	71.18

Supplementary Material S6: Characteristics of participants seronegative to all VPDs (measles, rubella, diphtheria, and tetanus).

Table S6: Overall (n=102) demographics of participants seronegative to all VPDs (measles, rubella, diphtheria, and tetanus).

Characteristic	Overall (n, %)
Age group	
5-9 years	65 (63.7%)
10+ years	37 (36.3%)
Year:	
2018	53 (51.9%)
2019	49 (48.1%)
Recruitment method:	
Household	49 (48.0%)
Convenience	53 (52.0%)
Gender:	
Female	54 (53.0%)
Male	48 (47.0%)

Figure S3: Map of primary sampling unit (PSU) level seroprevalence estimates[a] (%) for seronegativity to all vaccine preventable diseases (measles, rubella, diphtheria, and tetanus) in Samoa 2018 & 2019. This figure was created using ArcGIS Pro (Version 3.1.0).

[a] - Adjusted for study design and standardised to age and sex

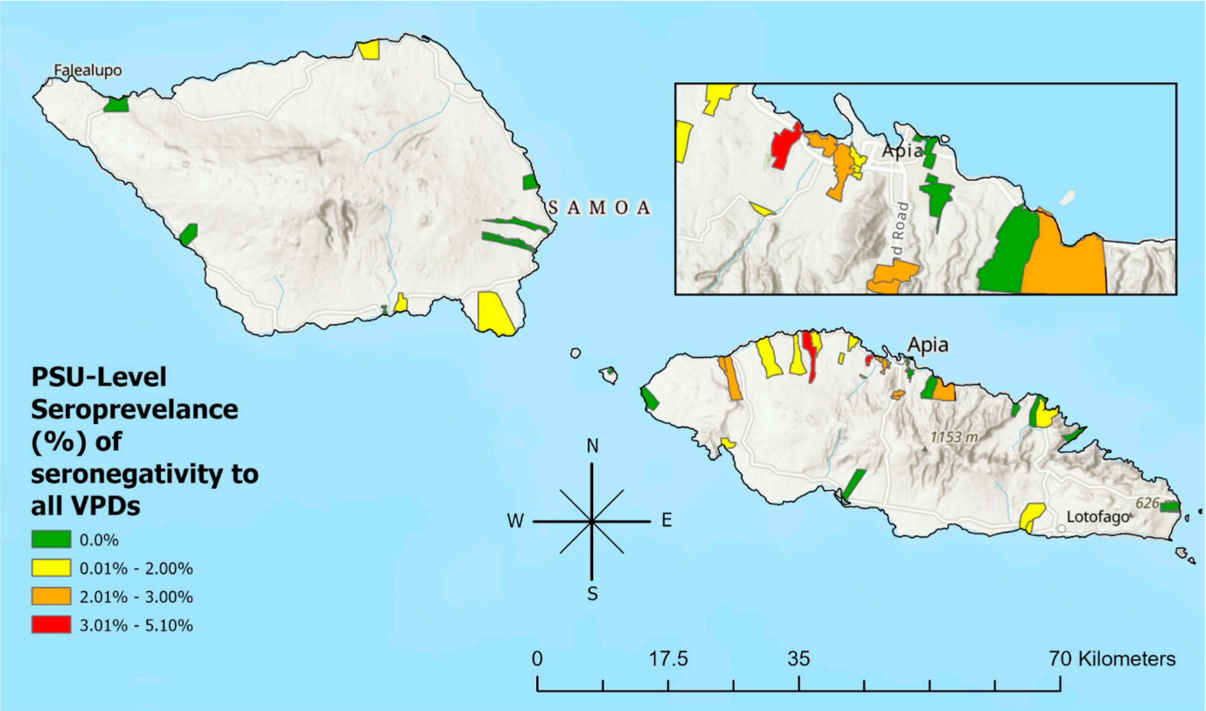

Figure S4: Map of household GPS coordinates and sampled primary sampling units.

This figure was created using ArcGIS Pro (Version 3.1.0).

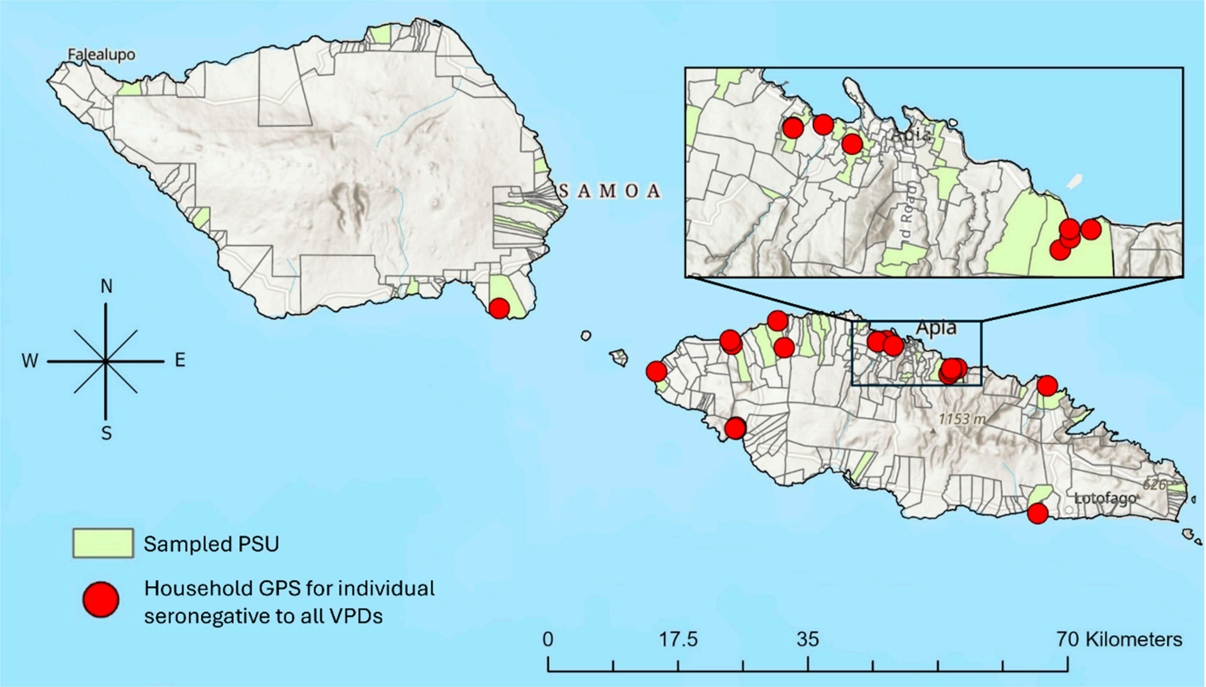

Figure S5: Overlap of Map of primary sampling unit (PSU) level seroprevalence estimates[a] (%) for seronegativity to all vaccine preventable diseases (measles, rubella, diphtheria, and tetanus) with household GPS coordinates and spatial cluster of seronegativity to measles.

This figure was created using ArcGIS Pro (Version 3.1.0).

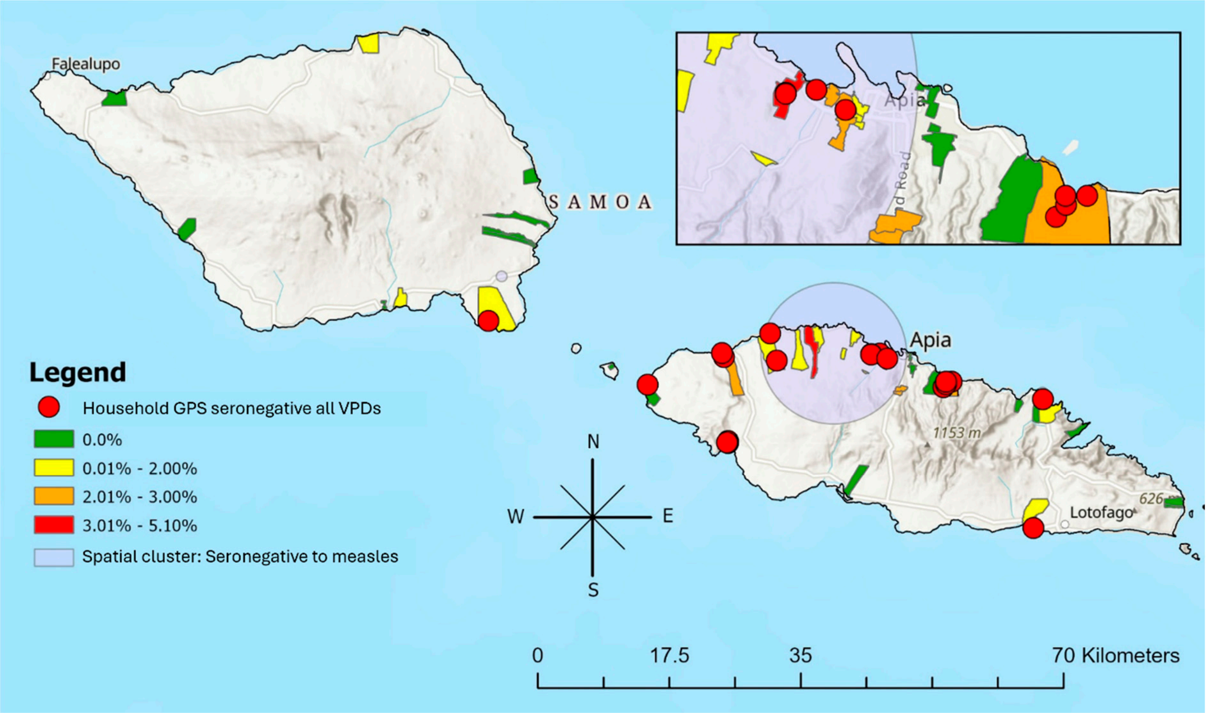

Supplement: Supplementary file 1 [file tropicalmed-11-00009-s001.zip › tropicalmed-4027418-supplementary.pdf]
